# Adiponectin reduces ER stress-induced apoptosis through PPAR*α* transcriptional regulation of ATF2 in mouse adipose

**DOI:** 10.1038/cddis.2016.388

**Published:** 2016-11-24

**Authors:** Zhenjiang Liu, Lu Gan, Tianjiao Wu, Fei Feng, Dan Luo, Huihui Gu, Shimin Liu, Chao Sun

**Affiliations:** 1 College of Animal Science and Technology, Northwest A&F University, Yangling, Shaanxi, China; 2 School of Animal Biology, The University of Western Australia, Crawley, Western Australia, Australia

## Abstract

Adiponectin is a cytokine produced predominantly by adipose tissue and correlates with glucose and lipid homeostasis. However, the effects of adiponectin on endoplasmic reticulum (ER) stress and apoptosis of adipose tissue remain elusive. In this study, we found that tunicamycin-induced ER stress increased serum free fatty acid (FFA) and impaired glucose tolerance, elevated the mRNA levels of *GRP78*, *Chop*, *ATF2* and *caspase 3*, but reduced *adiponectin* mRNA level in white adipose tissue. Moreover, ER stress-triggered adipocyte apoptosis by increasing cellular FFA level and Ca^2+^ level. Further analysis revealed that adiponectin alleviated ER stress-induced adipocyte apoptosis by elevating peroxisome proliferator-activated receptor alpha (*PPARα*) mRNA level. Our data also confirmed that adiponectin reduced early apoptotic cells and blocked the mitochondrial apoptosis pathway by activating the AdipoR1/AMP-activated protein kinase (AMPK) signal pathway. In addition, PPAR*α* bound to ATF2 promoter region and inhibited transcription of ATF2. The inhibition of adipocyte apoptosis by adiponectin was correlated with transcriptional suppression of ATF2. Furthermore, adiponectin inhibited ER stress-induced apoptosis by activating the AMPK/PKC pathway. In summary, our data demonstrate adiponectin inhibited ER stress and apoptosis of adipocyte *in vivo* and *in vitro* by activating the AMPK/PPAR*α*/ATF2 pathway. Our study establishes that adiponectin is an important adipocytokine for preventing and treating obesity.

Obesity has become a worldwide human health problem. Studies have demonstrated that the pathogenesis of obesity, type 2 diabetes and atherosclerosis is correlated with decreased plasma adiponectin level.^1^ Adiponectin is a hormone secreted by adipocytes, and regulates glucose homeostasis and lipid metabolism.^2, 3^ Recent studies show that adiponectin prevents the pathogenesis of cancer and Alzheimer's disease.^4, 5^ Our previous studies have shown that adiponectin inhibited pre-adipocyte differentiation and promoted mitochondrial biogenesis in mice and chicken.^6, 7^ Moreover, adiponectin regulated sodium intake and enhanced cold-induced browning of white adipose tissue (WAT).^8^ Adiponectin also protects cells from undergoing apoptosis and reduces inflammation in hepatocytes, endothelial cells and pancreatic beta cells.^9, 10^ However, the effects of adiponectin on adipocyte apoptosis have not been established.

Endoplasmic reticulum (ER) has a central role in protein synthesis, folding and transportation. It also serves as a key site for integrating cellular responses to stresses.^11, 12^ The ER stress response, also known as the unfolded protein response (UPR), involves translational attenuation, transcriptional induction of chaperones and folding enzymes, as well as degradation of misfolded proteins.^13^ Recent studies show that ER stress induces lipogenesis and promotes obesity-induced insulin resistance, type 2 diabetes and hepatic steatosis.^13, 14^ Current evidence suggests that the interaction between ER and mitochondria has important roles in oxidative stress and metabolic homeostasis.^15, 16^ Moreover, ER stress induces apoptosis in cancer cells and hematopoietic stem cells.^17, 18^ However, the relationship between ER stress and adipocyte apoptosis is still unclear.

Apoptosis, or programmed cell death, is essential for maintaining cellular homeostasis. Recently, the induction of apoptosis has been proposed as a newly alternative approach to prevent and treat obesity.^19^ Adipose tissue secretes abundant adipokines that affect the regulation of adipose weight and homeostasis,^20^ and several adipokines and natural products have roles in the induction of adipocyte apoptosis.^21, 22^ Our previous studies also showed that protein kinases or transcription factors had important roles in the regulation of adipocyte apoptosis.^23, 24, 25^ Among these kinases, AMP-activated protein kinase (AMPK) acts as a critical sensor in cellular energy homeostasis, and is also involved in the control of adipocyte apoptosis.^26^ The function of adiponectin is known for activating AMPK signal by adiponectin receptors 1 and 2.^27^ Therefore, the objective of this study was to elucidate the regulatory mechanisms of adiponectin on adipocyte apoptosis.

In this study, we demonstrated that adiponectin protected ER stress-induced apoptosis in adipose tissue, and confirmed that adiponectin worked through the AMPK/PKC pathway and transcriptional suppression of ATF2. The results imply that adiponectin could be used as a new therapeutic agent for preventing and treating obesity and type 2 diabetes.

## Results

### ER stress decreased adiponectin level in adipose tissue

**Figure 1:**
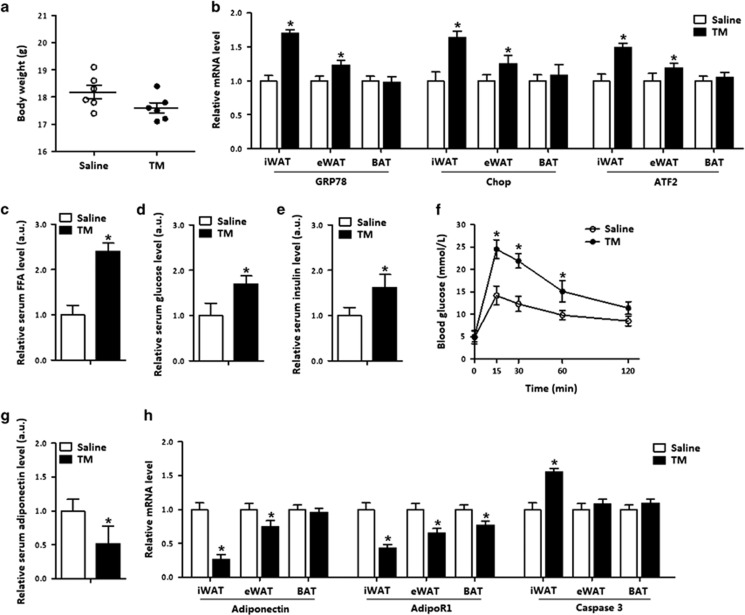
ER stress decreased adiponectin level in adipose tissue. The effects of ER stress on mice introduced by an injection of 1 *μ*g/g TM for 24 h are compared with an injection of saline (control), *n*=6 for each treatment. (**a**) Body weight of male mice. (**b**) Relative mRNA levels of *GRP78*, *Chop* and *ATF2* in iWAT, eWAT and BAT. (**c**) Serum FFA concentration. (**d**) Serum glucose concentration. (**e**) Serum insulin concentration. (**f**) GTT in mice. (**g**) Serum adiponectin level. (**h**) Relative mRNA levels of *adiponectin*, *AdipoR1* and *caspase 3* in adipose tissues. Values are means±S.D. *P<0.05 compared with the saline control

To investigate the effects of adiponectin on ER stress in adipose tissue, we injected tunicamycin (TM) intraperitoneally into mice. The body weight of mice was reduced after TM injection (Figure 1a). *GRP78* and *Chop* were increased in both inguinal WAT (iWAT) and epididymal WAT (eWAT), but not in brown adipose tissue (BAT); and along with the elevation of ATF2 in WAT but not in BAT (Figure 1b). The TM injection elevated serum free fatty acid (FFA) level, serum glucose and insulin levels (Figures 1c–e) but impaired body glucose tolerance and reduced insulin sensitivity in iWAT (Figure 1f and Supplementary Figure S1b). Stress also decreased serum adiponectin level and the mRNA level of *adiponectin receptor 1* (*adipoR1*) in WAT and BAT (Figures 1g and h). The *caspase 3* level was increased along with the elevated *Chop* expression, indicating that TM-induced ER stress led to adipose apoptosis (Figures 1b and h). In addition, the increased protein levels of IL-6, MCP-1 and TNF*-α* suggested TM-induced inflammation in iWAT (Supplementary Figure S1a).

We then used high-fat diet (HFD) induced obese mice to confirm the effects of TM-induced ER stress on adipose apoptosis. HFD feeding increased body weight, whereas TM treatment had the opposite result (Supplementary Figure S2a). Serum adiponectin level was reduced in the HFD group with or without the TM injection (Supplementary Figure S2b). As expected, HFD triggered ER stress and TM injection aggravated the ER stress response (Supplementary Figure S2c). The further measurement of inflammatory indices showed that HFD mice had increased levels of IL-6, MCP-1 and TNF*-α*, which increased slightly in response to the TM injection, although it was not significant (Supplementary Figure S2d). Interestingly, HFD feeding did not trigger adipose apoptosis; on the contrary, the TM injection markedly elevated the levels of *caspase 3* and *Bax* (Supplementary Figure S2e). Overall, TM-induced ER stress causes apoptosis in adipose tissue, and adiponectin and ATF2 may contribute to the disturbance of fatty acid metabolism and adipose apoptosis *in vivo*.

### Adiponectin reduced serum FFA and prevented ER stress in adipose tissue

**Figure 2:**
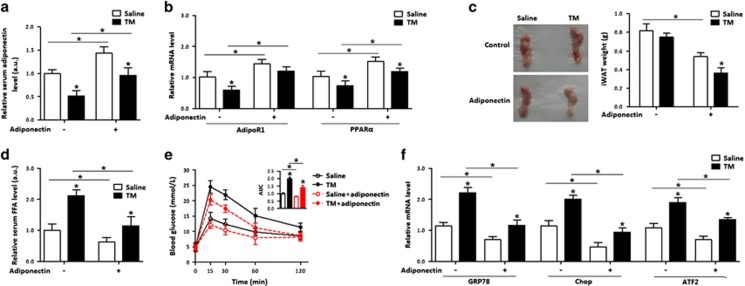
Adiponectin reduced serum FFA and prevented ER stress in adipose tissue. In all, 1 mg/kg recombinant murine adiponectin was injected into the tail vein of mice with or without ER stress. Injection of saline was used as control. *n*=6 for each treatment. (**a**) Serum adiponectin concentration. (**b**) Relative mRNA levels of *AdipoR1* and *PPARα*. (**c**) The iWAT weight of mice. (**d**) Serum FFA concentration. (**e**) GTT of mice. (**f**) Relative mRNA levels of *GRP78*, *Chop* and *ATF2*. Values are means±S.D. *P<0.05 compared with the saline control

We next addressed whether adiponectin elicited beneficial effects on apoptosis and disturbance of fatty acid metabolism. As shown in Figure 2a, the injection of recombinant adiponectin into mice under TM condition led to an increase of *AdipoR1,* which was decreased by the TM injection (Figure 2b). Compared with the TM treatment, adiponectin injection lowered iWAT weight (Figure 2c) and serum FFA level, and improved glucose tolerance (Figures 2d and e). Those changes were correlated with the reductions of *GRP78*, *Chop* and *ATF2* mRNA (Figure 2f). Interestingly, the expression of peroxisome proliferator-activated receptor alpha (*PPARα*) in adipose tissue was inhibited by the TM injection, but was elevated by the adiponectin injection (Figure 2b). These data collectively suggest that adiponectin is associated with ATF2 in ER stress-induced apoptosis in adipose tissue.

### Adiponectin relieved adipocyte ER stress and promoted fatty acid metabolism

**Figure 3:**
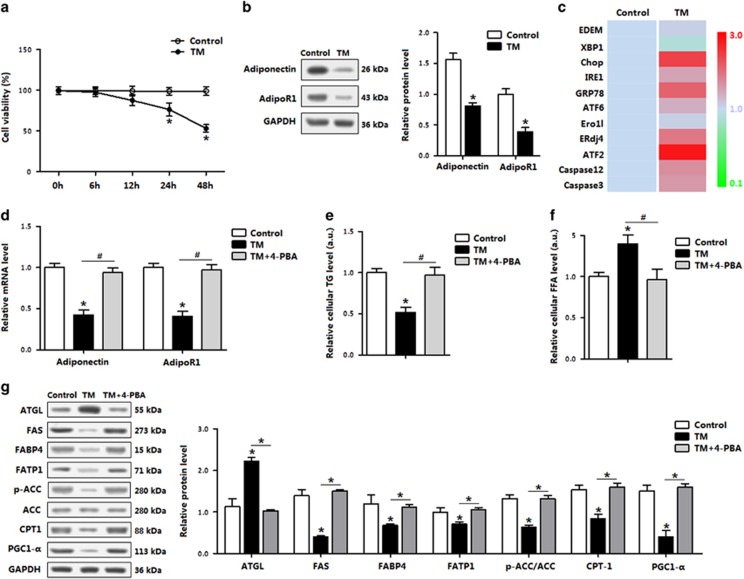
TM-induced ER stress, reduced adiponectin and impaired fatty acid metabolism in adipocytes. (**a**) Cell viability of adipocytes treated with TM (1 *μ*g/ml) for up to 48 h (*n*=3). (**b**) Protein levels of adiponectin and AdipoR1 in adipocytes incubated with TM for 12 h (*n*=3). (**c**) Relative mRNA levels of the ER stress genes in relevance to *β*-actin in adipocytes incubated with TM for 12 h (*n*=3). (**d**) Relative mRNA levels of *adiponectin* and *AdipoR1* in adipocytes incubated with TM for 12 h, followed with 4-PBA for 1 h (*n*=3). (**e**) TGs in adipocytes incubated first with TM for 12 h, then with 4-PBA for 12 h (*n*=3). (**f**) FFA level in adipocytes incubated with TM for 12 h, followed with 4-PBA for 1 h (*n*=3). (**g**) Protein levels relating fatty acid metabolism relative to GAPDH in adipocytes incubated first with TM for 12 h, then with 4-PBA for 1 h (*n*=3). Values are means±S.D. *P<0.05 compared with the control group

The effects of adiponectin on ER stress-triggered apoptosis in *in vitro* model are shown in Figure 3. The TM treatment for 12 h did not reduce cell viability (Figure 3a), but decreased levels of adiponectin and AdipoR1 (Figure 3b). Consistent with *in vivo* study data, TM-induced ER stress as indicated by the increases of *GRP78*, *ATF6*, *EDEM*, *XBP1*, *IRE1* and *ERdj4*, and adipocyte apoptosis was also triggered by elevated *Chop*, *caspase 3* and *caspase 12* (Figure 3c). *ATF2* was drastically increased under the ER stress condition (Figure 3c). When the ER stress in adipocytes was inhibited by 4-PBA, *adiponectin* and *AdipoR1* levels were recovered to those in control (Figure 3d), cellular triglycerides (TGs) concentration was elevated, whereas cellular FFA was reduced (Figures 3e and f). Western blot analysis of the key proteins for fatty acid transportation showed that the ER stress reduced fatty acid transportation, whereas blocking ER stress by using 4-PBA restored fatty acid transportation (Figure 3g).

**Figure 4:**
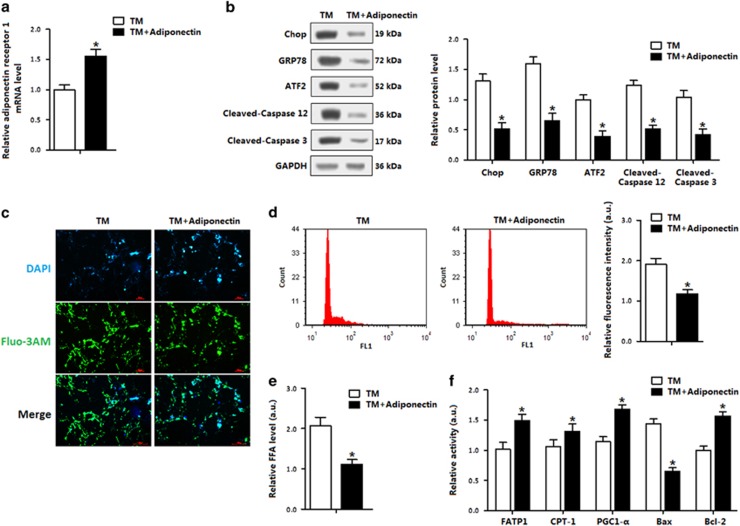
Adiponectin alleviated adipocyte ER stress and reduced cytosolic Ca^2+^. Adipocytes were first incubated with TM; recombinant murine adiponectin was then added to the medium at 10 *μ*g/ml. *n*=3 for each treatment. (**a**) Relative *AdipoR1* level in adipocytes. (**b**) Protein levels of ER stress and apoptosis. (**c**) Cytosolic Ca^2+^ in adipocytes. (**d**) Flow cytometry (FCM) analysis of Cytosolic Ca^2+^ in Figure 4c. (**e**) FFA level in adipocytes. (**f**) Protein levels of FATP1, CPT-1, PGC-1-*α*, Bax and Bcl-2 in adipocytes measured by using ELISA method. Values are means±S.D. *P<0.05 compared with the control group

In the stressed conditions, adiponectin elevated the expression of *AdipoR1* and decreased TM-induced ER stress and apoptosis in adipocytes (Figures 4a and b). Elevation of cytosolic Ca^2+^, an indicator of ER stress-induced apoptosis,^28, 29^ was decreased after the TM pretreatment (data not shown). Adiponectin incubation decreased the intracellular Ca^2+^ level (Figures 4c and d). These effects of adiponectin were similar to that of the 4-PBA. Cellular FFA concentration was reduced after adiponectin addition, along with increased fatty acid transportation and prevention of adipocyte apoptosis (Figures 4e and f). Thus, the data in Figures 2,3 and 4 support the hypothesis that adiponectin attenuates ER stress and apoptosis, and promotes fatty acid transportation.

### Adiponectin blocked PA-induced apoptosis and decreased ATF2

**Figure 5:**
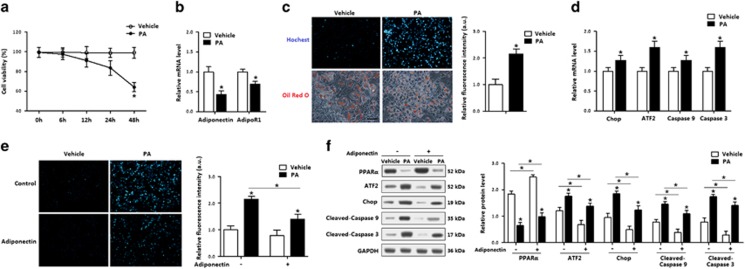
Adiponectin blocked PA-induced apoptosis and decreased ATF2 in adipocytes. Recombinant murine adiponectin was added in the medium at 10 *μ*g/ml. *n*=3 for each treatment. (**a**) Cell viability of adipocytes treated with palmitic (200 *μ*g/ml, PA) for up to 48 h. (**b**) Relative mRNA levels of *adiponectin* and *AdipoR1* in adipocytes incubated with PA for 24 h. (**c**) Hoechst and Red Oil O-stained adipocytes incubated with PA for 24 h. (**d**) Relative mRNA levels of *Chop*, *ATF2*, *caspase 9* and *caspase 3* in adipocytes incubated with PA for 24 h. (**e**) Hoechst-stained adipocytes incubated with PA, followed with adiponectin (10 *μ*g/ml). (**f**) Protein levels of PPAR*α*, ATF2, Chop, cleaved-caspase 9 and cleaved-caspase 3 in adipocytes incubated with PA, followed with adiponectin (10 *μ*g/ml). Values are means±S.D. *P<0.05 compared with the control group

FFA is mainly catabolized in mitochondria, and the disturbance of fatty acid metabolism triggers mitochondria-related apoptosis. To explore whether adiponectin mitigated the ER stress and apoptosis via the regulation of mitochondrial functions, we used palmitate (PA)-induced apoptosis in *in vitro* model. Cell viability measurement indicated the PA treatment for 24 h did not affect cell viability (Figure 5a). PA reduced the mRNA levels of both *adiponectin* and *AdipoR1* (Figure 5b), caused adipocyte apoptosis, which was accompanied with the elevation of *ATF2* (Figures 5c and d). Then Hoechst staining analysis showed adiponectin markedly reduced PA-induced adipocyte apoptosis (Figure 5e). Classical apoptosis proteins were consistently decreased with the adiponectin incubation, and ATF2 protein level was also reduced (Figure 5f). Hence, the anti-apoptosis function of adiponectin in PA-induced mitochondrial apoptosis was confirmed, and ATF2 was involved in this process.

### Adiponectin inhibited mitochondrial apoptosis pathway and reduced ER stress in adipocytes

**Figure 6:**
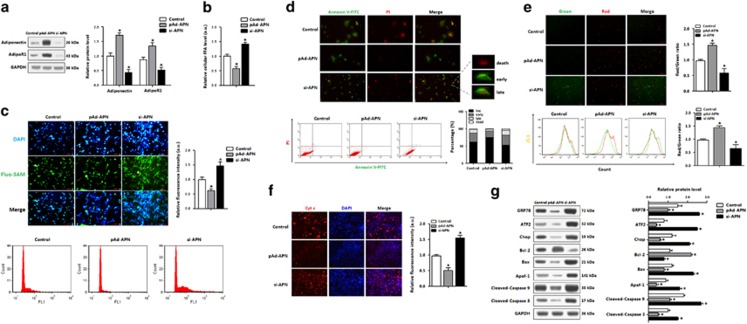
Adiponectin inhibited mitochondrial apoptosis pathway and reduced ER stress in adipocyte. Adipocytes were infected with recombinant vectors of adiponectin (pAd-APN or si-APN) for 48 h. *n*=3 for each treatment. (**a**) Protein levels of adiponectin and adipoR1 in adipocytes. (**b**) FFA concentration in adipocytes. (**c**) Cytosolic Ca^2+^ detected by using Fluo-3-AM stain and flow cytometry (FCM) analysis of fluorescence intensity in adipocytes. (**d**) Annexin V-FITC/PI double staining and flow cytometry analysis of adipocyte apoptosis stages. (**e**) JC-1 staining and flow cytometry analysis of mitochondrial membrane potential in adipocytes. (**f**) Cyt C immunofluorescence in adipocytes. (**g**) Protein levels of ATF2 and apoptosis genes in adipocytes. pAd-APN, recombinant adenovirus overexpression vector of adiponectin; si-APN, recombinant lentiviral interference vector of adiponectin. Values are means±S.D. *P<0.05 compared with the control group

To explore how adiponectin reduced mitochondrial apoptosis, the recombinant virus vectors of adiponectin was used in adipocytes, and the optimum infection efficiency was shown in Figure 6a. Forced expression of adiponectin reduced serum FFA (Figure 6b). In an absence of adiponectin, intracellular Ca^2+^ was trapped in cytoplasm, whereas adiponectin overexpression showed the opposite results (Figure 6c). As loss of membrane integrity can cause a disturbance of Ca^2+^ influx and lead to apoptosis, our data showed mitochondrial membrane potential was elevated in the pAd-APN treatment (Figure 6e). Cyt C was reduced by adiponectin, whereas in the absence of adiponectin, the membrane potential was decreased and Cyt C was increased (Figure 6f). Moreover, adiponectin reduced the number of early- and late-stage apoptotic cells (Figure 6d). At the protein level, the adiponectin treatment increased the Bcl-2 protein level, but lowered the levels of GRP78, Chop, Bax, Apaf-1, cleaved-caspase 3/9 proteins (Figure 6g). The ATF2 level was markedly decreased (Figure 6g). Therefore, adiponectin was involved in the regulation of ER stress and hence alleviated mitochondrial apoptosis.

### PPAR*α* inhibited transcription of ATF2 and ER stress-induced apoptosis of adipocytes

**Figure 7:**
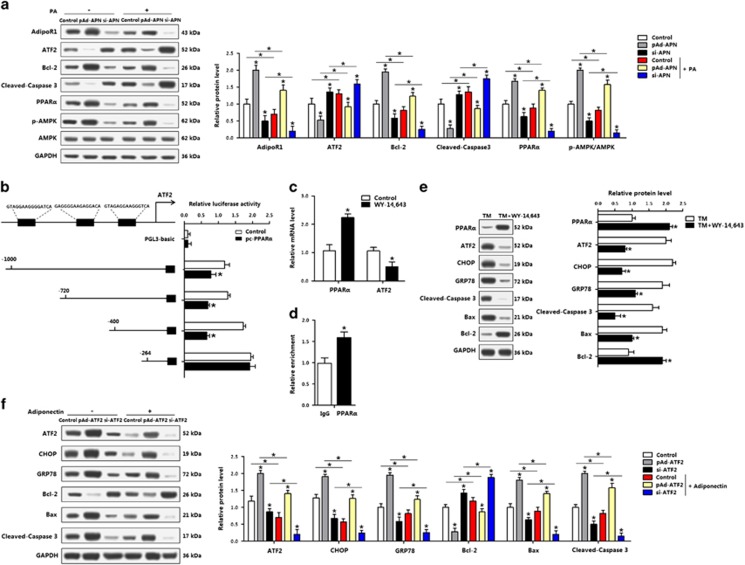
PPAR*α* inhibited transcription of ATF2 and ER stress-induced apoptosis of adipocytes. (**a**) Protein levels of adipoR1, ATF2, Bcl-2, cleaved-caspase 3 and PPAR*α*, p-AMPK in adipocytes infected with pAd-APN or si-APN and incubated with PA (*n*=3). (**b**) Dual-luciferase reporter assay of ATF2 and PPAR*α*. Cells were transfected with PGL3-basic or PGL3-ATF2 plasmids, and pc-PPAR*α* plasmid (*n*=3). (**c**) mRNA levels of *PPARα* and *ATF2* after WY-14 643 treatment (*n*=3). (**d**) ChIP analysis between ATF2 and PPAR*α* (*n*=3). (**e**) Protein levels of PPAR*α*, ATF2, CHOP, GRP78, cleaved-caspase 3, Bax and Bcl-2 incubated with TM, followed with WY-14  643 (*n*=3). (**f**) Protein levels of ATF2, CHOP, GRP78, Bcl-2, Bax and cleaved-caspase 3 of adipocytes infected with pAd-ATF2 or si-ATF2, then incubated with adiponectin (*n*=3). pAd-APN, recombinant adenovirus overexpression vector of adiponectin; si-APN, recombinant lentiviral interference vector of adiponectin. pAd-ATF2, recombinant adenovirus overexpression vector of ATF2; si-ATF2, recombinant lentiviral interference vector of ATF2. Values are means±S.D. *P<0.05 compared with the control group

The AMPK signal pathway was examined in adipocytes pretreated with PA. Adiponectin activated AMPK during mitochondrial apoptosis (Figure 7a), and ATF2 and PPAR*α* showed the opposite expression profiles (Figure 7a). As PPAR*α* is downstream of AdipoR1, we next examined whether adiponectin reduced apoptosis via the regulation of PPAR*α* and ATF2. Our data showed PPAR*α* bound to the promoter region of ATF2, and reduced ATF2 transcription (Figures 7b and d). In addition, by using the PPAR*α* agonist, WY-14 663, the mRNA expression of ATF2 was blunted (Figure 7c). The WY-14 643 led to the reduction of ATF2, along with decreased protein levels of CHOP, GRP78, cleaved-caspase 3 and Bax (Figure 7e). Overexpression of ATF2 promoted adipocyte apoptosis, which was alleviated by an addition of adiponectin, indicating that ATF2 had the opposite effects as PPAR*α* and adiponectin in reducing adipocyte apoptosis (Figure 7f). Together, PPAR*α* regulated ATF2 in the ER stress of adipocytes, and had an anti-apoptosis role.

### AMPK/PKC signal was essential for adiponectin inhibited apoptosis of adipocytes

**Figure 8:**
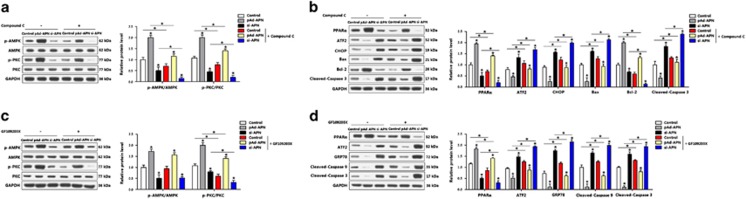
AMPK/PKC signal was essential for adiponectin inhibited apoptosis of adipocytes. (**a**) Protein levels of p-AMPK and p-PKC of adipocytes infected with pAd-APN or si-APN for 48 h, then incubated with compound C (*n*=3). (**b**) Protein levels of PPAR*α*, ATF2, CHOP, Bax, Bcl-2 and cleaved-caspase 3 of adipocytes infected with pAd-APN or si-APN, then incubated with compound C (*n*=3). (**c**) Protein levels of p-AMPK and p-PKC of adipocytes infected with pAd-APN or si-APN, then incubated with GF109203X (*n*=3). (**d**) Protein levels of PPAR*α*, ATF2, GRP78, cleaved-caspase 9 and cleaved-caspase 3 of adipocytes pre-infected with pAd-APN or si-APN, incubated with GF109203X (*n*=3); pAd-APN, recombinant adenovirus overexpression vector of adiponectin; si-APN, recombinant lentiviral interference vector of adiponectin. Values are means±S.D. *P<0.05 compared with the control group

The functions of adiponectin on the regulation of AdipoR1 and AMPK signals in adipocytes were examined by measuring p-AMPK and p-PKC with or without compound C, and the results were shown in Figure 8a. Knockdown of adiponectin reduced the levels of both p-AMPK and p-PKC, whereas addition of compound C intensely blocked the activity of both AMPK and PKC (Figure 8a). Overexpression of adiponectin elevated the protein levels of PPAR*α*, and reduced ATF2 and adipocyte apoptosis. Compared with pAd-APN group, pretreatment of adipocytes with adiponectin and compound C reduced PPAR*α*, increased ATF2 and apoptosis genes (Figure 8b). When PKC was inactivated by using GF109203, the PKC activity was blocked, but the activity of AMPK was not affected (Figure 8c), suggesting PKC is downstream of AMPK. Inactivating PKC also elevated ATF2 and promoted apoptosis. Addition of adiponectin restored the activity of PKC, antagonized the effects of GF109203 and decreased cell apoptosis (Figure 8d). These data suggest effect of adiponectin in reducing adipocyte apoptosis is through the AMPK/PKC signal pathway.

## Discussion

Understanding cellular responsive mechanisms of adipocytes during the development of obesity is essential in order to develop preventative and treatment strategies. Stress-related alterations in ER, such as the UPR, are associated with obesity.^30^ The ER stress leads to insulin resistance, augments lipolysis and triggers inflammation in adipose tissue. In this study, we confirmed that TM-induced ER stress and inflammation in adipose tissue of both healthy and obese mice. However, inflammation is not responsible for ER stress-induced adipose apoptosis.^31, 32, 33^ Moreover, ER stress also induces lipotoxic and dysfunction in pancreatic *β* cells and macrophages.^34, 35^ Our results show that ER stress reduced adiponectin levels in both serum and adipose tissue. These findings are consistent with published reports that demonstrate adiponectin is closely related to ER stress in adipocytes.^36, 37^ The disturbance of intracellular Ca^2+^ signal has an important role in connecting ER stress to mitochondrial functions.^15, 38^ As mitochondrial Ca^2+^ overload alerts the morphology of mitochondria, and triggers cell apoptosis.^39, 40^ Recent work demonstrates that adiponectin induces a remarkable Ca^2+^ influx in the skeletal muscle to control mitochondrial biogenesis.^41, 42^ Here our data demonstrate that ER stress disturbed Ca^2+^ distribution, inhibited cellular FFA metabolism and caused apoptosis in adipocyte. Moreover, adiponectin alleviated the cellular Ca^2+^ disturbance and eliminated ER stress-induced FFA deposition in adipocytes. These results facilitate our understanding of the relationships between ER stress and mitochondrial apoptosis, although the interaction between ER and mitochondria has been studied extensively.^43, 44^

Adiponectin is an adipocyte-specific factor, which have beneficial effects on obesity, diabetes, inflammation, atherosclerosis and cardiovascular diseases. Adiponectin protects against liver tumorigenesis through increasing apoptosis of hepatocellular carcinoma cells. Many cancer cell lines express adiponectin receptors, so adiponectin limits cancer cell proliferation and induce apoptosis.^5, 45, 46^ However, adiponectin also inhibits apoptosis of cardiac cells, mesenchymal stem cells and auditory hair cells.^47, 48, 49^ Thus, the ability of adiponectin to mediate an alternative function in cell apoptosis provides an attractive hypothesis to explain its protective effects in apoptosis of adipocytes. In this study, we showed that adiponectin protects ER stress-induced apoptosis of adipocytes, and adiponectin also reduces Cyt C releases to cytoplasm, Cyt C then inhibited the mitochondrial apoptotic pathway. Thus, our results suggest that adiponectin inhibits adipocyte apoptosis by modulating the interaction between ER and mitochondria, and furthermore this inhibitory effect is through activating the adipoR1/AMPK pathway. These findings are consistent with literatures that adiponectin activates its receptors, adipoR1 and adipoR2, then increases the activity of AMPK to exert its function,^50, 51^ which leads to detailed studies on the crystal structures and small-molecule agonist of adipoR1 and adipoR2.^52, 53^ Consequently, more molecular mechanisms on the adiponectin signal in adipocytes apoptosis warrant further studies.

PPAR*α*, downstream of adipoR1 and adipoR2 signals, has a critical role in signal transduction and transcriptional regulation in adipocyte.^54, 55^ PPAR*α* also regulates proliferation and apoptosis in other cell types.^56^ We showed in this study that adiponectin inhibited FFA-induced apoptosis by elevating the PPAR*α* expression in mouse adipocytes. ATF2 is a member of the activator protein-1 complex, and has important roles in cellular stress responses in many cell types.^57, 58^ ATF2 activates the CHOP pathway during ER stress and induces cell apoptosis.^59^ Moreover, the PKC pathway regulates ATF2 and attenuates apoptosis in tumor-suppressing functions.^60^ Our data in this study suggest that adiponectin inhibited ER stress-induced adipocyte apoptosis via activating the AMPK/PKC pathway to reduce ATF2 expression, demonstrating that ATF2 is essential for the inhibitory effect of adiponectin on adipocyte apoptosis. Other studies have shown that PPAR*α* promotes fatty acid oxidation and reduces FFA level by regulating genes involved in the transport and degradation of fatty acids.^61, 62^ In addition, ATF2 promotes lipolysis and increases FFA level, indicating ATF2 has the opposite function compared with PPAR*α*.^63, 64^ Although few studies have established the relationship between PPAR*α* on ATF2, we postulated that PPAR*α* directs the regulation of ATF2.^65^ Interestingly, we showed that PPAR*α* binds to the ATF2 promoter region resulted in inhibition of ATF2 transcription, suggesting that adiponectin inhibited adipocyte apoptosis through promoting PPAR*α* transcriptional inhibition of ATF2. It is likely that PPAR*α* has a key role in regulating the crosstalk between ER and mitochondria.

In summary, our data provide compelling evidence that adiponectin inhibits ER stress-induced apoptosis through the AdipoR1/AMPK/PKC pathway. Moreover, we found that PPAR*α* was a novel transcriptional suppressor of ATF2 in alleviating ER stress and apoptosis of adipocytes (Supplementary Figure S3). Our results contribute to further understanding of regulatory mechanisms of adipocytes apoptosis for the development of novel approaches to prevent and treat obesity.

## Materials and Methods

### Animal experiment

Eight-week-old C57BL/6J male mice were purchased from the Laboratory Animal Center of the Fourth Military Medical University (Xi'an, China). Mice handling protocols were conducted following the guidelines and regulations approved by the Animal Ethics Committee of Northwest A&F University. Mice were provided *ad libitum* water and a standard laboratory chow diet purchased from Animal Center of the Fourth Military Medical University. In diet-induced obesity study, mice were placed on HFD (fat provides 60% of the total energy) for 10 weeks, whereas control mice were fed with a standard chow diet (fat provides 10% of the total energy). Body weight and food intake of mice were recorded weekly. The animal room was maintained at 25±1 °C, humidity at 55±5% and 12-h light–dark cycles.

The mice (*n*=24) were randomly divided into four groups using a 2 × 2 factorial design. Half of the mice were intraperitoneally injected with saline (control), and the other half injected with TM (1 *μ*g/g) 24 h before the last dark cycle. The TM injection was used to create ER stress in adipose tissue of mice. To address the effect of adiponectin on apoptosis of adipose tissue, half of the mice that received the saline or TM injection were injected with 1 mg/kg recombinant murine adiponectin (Peprotech, Rocky Hill, NJ, USA) into the tail vein of mice 6 h after the saline or TM injection. Mice were then killed by ethyl ether. The iWAT, eWAT and BAT were dissected, their weights recorded, and the tissues were used for the following studies.

### Metabolic phenotyping

Glucose tolerance test (GTT) in mice was carried out after TM treatment for 24 h, and mice were injected intraperitoneally 1.2 mg glucose/g body weight after overnight fasting. Blood glucose levels were assessed using the Accu-Chek glucose monitor (Roche Diagnostics Corp., Pleasanton, CA, USA) before the injection and then at 15, 30, 60 and 120 min post injection. Serum insulin and adiponectin levels were measured using ELISA kits from Abcam (Cambridge, UK), per supplier's protocols. Serum FFAs and glucose were measured using the Free Fatty Acid Quantification kit and Glucose Assay kit from Abcam, respectively.

The measurement of protein levels of IL-6, MCP-1 and TNF*-α* were conducted using the commercial ELISA kits from Abcam according to the manufacturer's instructions.

### Primary adipocyte culture

The connective fiber and blood vessels were removed from the iWAT, and washed three times with PBS buffer containing 200 U/ml penicillin (Sigma, St. Louis, MO, USA) and 200 U/ml streptomycin (Sigma). The adipocyte culture was carried out according to the protocols described in our previous publication.^66^ Briefly, pre-adipocytes were seeded onto 35-mm culture dishes at a density of 8 × 10^4^ cells per dish, and incubated at 37 °C under a humidified atmosphere of 5% CO_2_ and 95% air until confluence. Differentiation of pre-adipocytes was performed as follows. Cells grown to 100% confluence (day 0) were induced to differentiation using DMEM/F12 medium containing dexamethasone (1 *μ*M, Sigma), insulin (10 *μ*g/ml, Sigma), IBMX (0.5 mM, Sigma) and 10% FBS. Four days after the induction (from day 2), cells were maintained in the induction medium containing insulin (10 *μ*g/ml, Sigma) and 10% FBS until the day for cell harvesting. Oil Red O staining was conducted according to the protocol described in our previous publication.^66^

### Chemical treatment and vectors infection

An *in vitro* model was used to elucidate how adiponectin contributes to ER stress-triggered apoptosis in adipocytes of iWAT. Pre-adipocytes of iWAT were differentiated to mature adipocytes as described above, and then used for the tests below.

The adipocytes were incubated with TM (final concentration 1 *μ*g/ml) for 24 h to create ER stress on the cells. Various chemical treatments were then applied to the TM-treated cells as follows: (a) addition of 4-phenybutyrate (4-PBA, 5 mM) to inhibit the ER stress; (b) addition of recombinant murine adiponectin (10* μ*g/ml) to examine the effect of adiponectin on the ER stressed cells; (c) to induce an apoptotic model, PA (200 *μ*g/ml) or 0.5% BSA (control for the PA treatment) was added into adipocyte. All those treatments were applied in the last hour of the incubation.

The regulatory roles of adiponectin on the AMPK/PKC pathway in the adipocytes on the stress or stress-inhibited conditions were investigated using the model described above. Adipocytes were first infected with adenovirus and lentiviral recombinant vectors for 48 h at the titer of 1 × 10^9^ IFU/ml, and with recombinant adenovirus overexpression vectors of adiponectin and ATF2 (pAd-APN, pAd-ATF2) to test the effect of the changes of adiponectin expression on ATF2 and ER stress. The control vector was pAd-GFP. PPAR*α* agonist WY-14 643 was used to elevate PPAR*α*. Compound C (AMPK inhibitor) and GF109203X (PKC inhibitor) were used to inhibit the AMPK/PKC pathway.

The recombinant lentiviral interference vectors of adiponectin and ATF2 (si-APN, si-ATF2) and control vector (pGLVU6/GFP) were purchased from Gene Pharma (Shanghai, China), and the other vectors were kept in our laboratory. PPAR*α* agonist WY-14 643, AMPK inhibitor compound C and PKC inhibitor GF109203X were obtained from Sellect Chemicals, Houston, TX, USA. Cellular TG, FATP1, CPT-1, PGC-1*α*, Bax and Bcl-1 levels were measured using commercial enzyme-linked immunosorbent (ELISA) kits (R&D Systems, Minneapolis, MN, USA).

### Intracellular calcium measurement

The intracellular calcium level was measured by using a fluorescent dye Fluo-3 AM (Beyotime Institute of Biotechnology, Nanjing, China), which across the cell membrane and Fluo-3 formed under canalization by intracellular esterase. Fluo-3 specifically combines Ca^2+^, generating strong fluorescence with an excitation wavelength at 488 nm. After getting exposed to TM, pAd-APN or si-APN, adipocytes were harvested and washed twice with PBS, and resuspended in 500 *μ*l Fluo-3 AM (3 *μ*M) for 60 min in dark. Then the cells were washed twice with PBS and stained with DAPI for 5 min. Fluorescence intensity was measured by BD FACScan (BD Biosciences, Franklin Lakes, NJ, USA), and data were analyzed using Cell Quest software (BD Biosciences).

### Apoptosis assay

Cell viability was first measured using Cell Counting Kit-8 (CCK-8, Vazyme, Nanjing, China) assay after incubation with TM or PA.^23^ After treatment with PA, adipocytes were incubated for 30 min with Hoechst 33342 (Solarbio, Beijing, China) loading dye, and washed three times with ice-cold PBS. Mid-stage and late-stage apoptosis of adipocytes were assayed by using Annexin V-FITC/PI apoptosis detection kit (Beyotime Institute of Biotechnology) following the user protocol. At the end of the incubation time, the cells were gently washed with cold PBS and suspended in 500 ml Annexin V binding buffer. After staining twice with 5 ml FITC labeled-Annexin V and 5 ml PI, cells were incubated for 30 min at room temperature in dark. The cells were viewed immediately at room temperature with an inverted fluorescent microscope (Nikon TE2000-U, Tokyo, Japan), and analyzed by BD FACScan (BD Biosciences). Data were analyzed using Cell Quest software (BD Biosciences).

### Mitochondrial membrane potential

Mitochondrial membrane potential is an index to membrane integrity. Fluorescent probe JC-1 (Beyotime Institute of Biotechnology) was used to estimate the membrane potential. Adipocytes were incubated at 37 °C for 10 min with 5 *μ*g/ml JC-1, then washed twice with cold PBS and placed in fresh medium without serum. Images were scanned using a Fluorescence Microscope (Nikon TE2000-U), following the procedure as described in our previous publication. The ratio of red/green fluorescent intensity was then calculated after the flow cytometry measurement.

### Cyt C immunofluorescence analysis

Cyt C immunofluorescence analysis was performed as previously described.^23^ Adipocytes were fixed with 10% neutral formalin for 30 min, incubated with Cyt C (Abcam) for 12 h at 4 °C, and then incubated with fluorescein isothiocyanate-conjugated goat anti-rabbit IgG antibody (Boster, Wuhan, China) for 1 h at room temperature. Finally, the cells were photographed with an inverted fluorescent microscope (Nikon TE2000-U).

### Promoter reporter assay and dual-luciferase reporter assay

The ATF2 promoter sequence was analyzed using Genomatrix MatInspector (Genomatrix Software GmbH, Munich, Germany). Four fragments containing ATF2-5' sequences from −1000 to −264 relative to the transcription initiation site were sub-cloned into pGL3-basic vector (Takara, Dalian, China). HEK293 cells were cultured in 24-well plates till 80–90% confluence and co-transfected with Renilla plasmid, PGL3-Foxc2 or pGL3-basic plasmid (control reporter), and PPAR*α* overexpression plasmid (pc-PPAR*α*). Cells were harvested 36 h after transfection and detected using the Dual-Luciferase Reporter assay system (Promega, Madison, WI, USA).

### ChIP assay

Adipocytes were prepared for chromatin immunoprecipitation (ChIP) assay using a ChIP assay kit (Abcam) according to the manufacturer's protocol. Primary antibodies of PPAR*α* (Abcam) or IgG (Abcam) were used. DNA–protein crosslinking complexes were collected, and purified DNA was subjected to qPCR with SYBR green fluorescent dye (Invitrogen, Carlsbad, CA, USA).

### Total RNA extraction, cDNA synthesis and real-time PCR

Total RNA was extracted from adipose tissues or adipocytes using TRIpure Reagent kit (Takara) according to the manufacturer's instructions. In all, 500 ng of total RNA was reverse transcribed using M-MLV reverse transcriptase kit (Takara). Primers were synthesized by Invitrogen (Shanghai, China). Real-time PCR was carried out in StepOnePlus^TM^ System (Applied Biosystems, Carlsbad, CA, USA) with SYBR Green Master Mix (Vazyme, Nanjing, China). The 2^−△△Ct^ method was used to quantitate the relative changes in gene expression normalized to *β*-actin.

### Protein extraction and western blot analysis

Protein from adipocytes was extracted using lysing buffer. Protein concentration was determined using BCA Protein Assay kit (Beyotime Institute of Biotechnology). Proteins (30 *μ*g) were separated by SDS-PAGE, transferred to PVDF nitrocellulose membrane (Millipore, Boston, MA, USA), blocked with 5% fat-free milk for 2 h at room temperature and then incubated with primary antibodies in 5% milk overnight at 4 °C. Adiponectin (ab75989), adiponectin receptor 1 (ab126611), CHOP (ab179823), GRP78 (ab108615), ATF2 (ab47476), active-caspase 12 (ab18766), Bax (ab32503), Apaf-1 (ab32372), PPAR*α* (ab8934), PGC-1*-α* (ab72230), p-AMPK (ab133448), AMPK (ab32047), p-PKC (ab32502), PKC (ab179522), FAS (ab128870), ATGL (ab109251), FABP4 (ab92501), FATP1 (ab69458), IRS1 (ab52167), p-IRS1 (ab46800), Akt (ab8805), p-Akt (ab81283) and Glut4 (ab188317) antibodies were all purchased from Abcam. Active-caspase 3 (bs7004), active-caspase 9 (bs7070), Bcl-2 (bs1511) and GAPDH (ap0063) antibodies were purchased from Bioworld (Nanjing, China). p-ACC (11818), ACC (4190) and CPT-1 (12252) antibodies were purchased from Cell Signaling Technology (CST, Boston, MA, USA). Rabbit HRP-conjugated secondary antibody (Boaoshen, Beijing, China) was added and incubated at room temperature for 2 h. Proteins were visualized using chemiluminescent peroxidase substrate (Millipore), and then the blots were quantified using ChemiDoc XRS system (Bio-Rad, Hercules, CA, USA).

### Statistics

Statistical analyses were performed using SAS v8.0 (SAS Institute, Cary, NC, USA). Data were analyzed using one-way or two-way ANOVA procedure. Comparisons among individual means were made by Fisher's least significant difference. Data were presented as mean±S.D. *P*<0.05 was considered to be significant.
