# Supplementary material for: Adiponectin reduces ER stress-induced apoptosis through PPARα transcriptional regulation of ATF2 in mouse adipose
Source: Cell Death Dis. 2016 Nov 24;7(11):e2487–. doi: 10.1038/cddis.2016.388 (PMC5260871; doi:10.1038/cddis.2016.388)
Supplement: Supplementary Figures [file cddis2016388x1.docx]

**Adiponectin reduces ER stress-induced apoptosis through PPARα transcriptional regulation of ATF2 in mouse adipose**

Zhenjiang Liu, Lu Gan, Tianjiao Wu, Fei Feng, Dan Luo, Huihui Gu, and Chao Sun

**Figure S1**


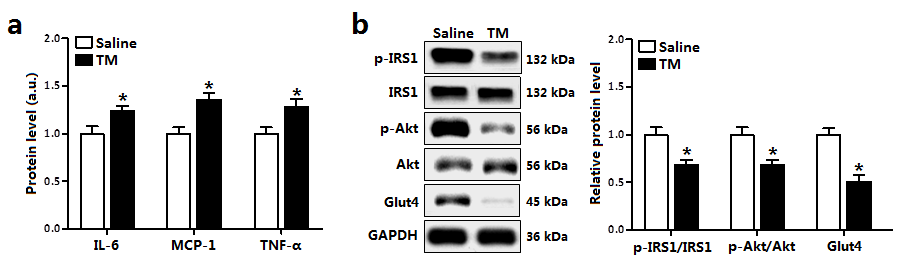


**Figure S1** (Related with Figure 1) (**a**) Protein levels of IL-6, MCP-1 and TNF-α of iWAT after TM injection. Measurement was conducted using ELISA method (n=6). (**b**) Insulin sensitivity and glucose tolerance of iWAT after TM injection (n=6). Values are means ± SD. * *p* < 0.05 compared with the saline control.

**Figure S2**


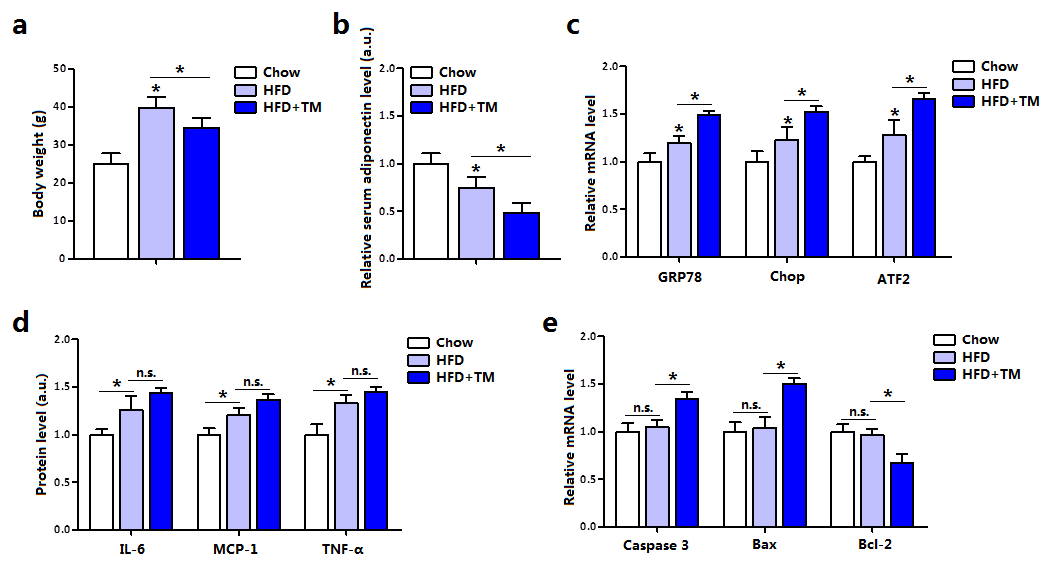


**Figure S2** (Related with Figure 1) (**a**) Body weight of mice with HFD or chow diet for 10 weeks, following injected with TM or not (n=6). (**b**) Serum adiponection level of mice with HFD or chow diet for 10 weeks, following injected with TM or not (n=6). (**c**) Relative mRNA levels of *GRP78*, *Chop* and *ATF2* of mice iWAT with HFD or chow diet for 10 weeks, following injected with TM or not (n=6). (**d**) Protein levels of IL-6, MCP-1 and TNF-α of mice iWAT with HFD or chow diet for 10 weeks, following injected with TM or not. Measurement was conducted using ELISA method (n=6). (**e**) Relative mRNA levels of *Caspase3*, *Bax* and *Bcl-2* of mice iWAT with HFD or chow diet for 10 weeks, following injected with TM or not (n=6). Values are means ± SD. * *p* < 0.05 compared with the saline control.

**Figure S3**


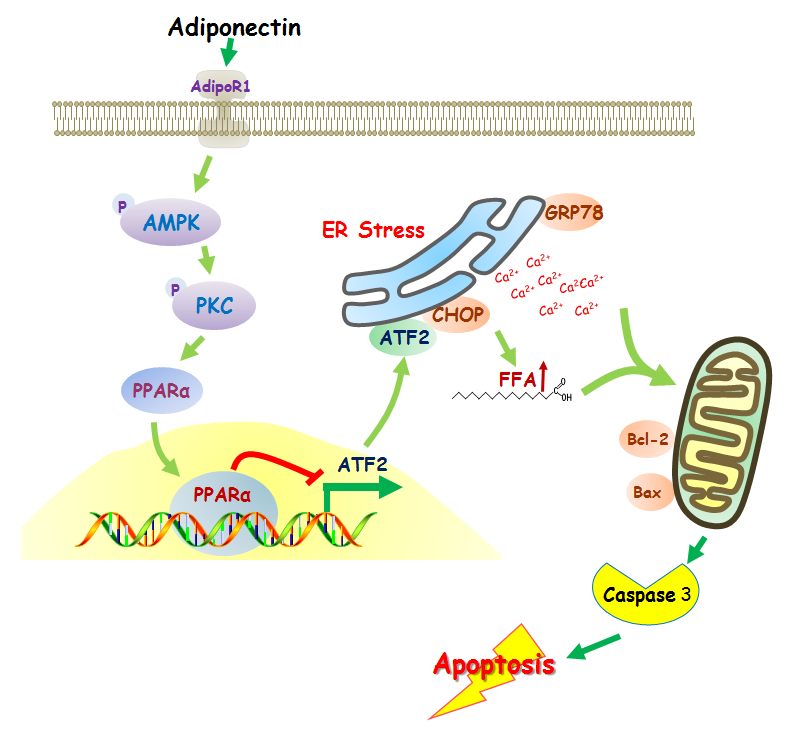


**Figure S3** Adiponectin inhibits ER stress-induced apoptosis through the AdipoR1/AMPK/PKC pathway. PPARα is a novel transcriptional suppressor of ATF2 in alleviating ER stress and apoptosis of adipocyte.
